# Parliament and the Professions

**Published:** 1919-12-06

**Authors:** 


### PARLIAMENT AND THE PROFESSIONS.

### Registration of Nurses.

In answer to a question as to whether it is proposed to put upon the Council under the Bill a representative of the Poor-law nursing services, Sir T. Walters said that he could make no statement, but he was alive to the importance of securing that the Council shall be as representative as possible of all types of nursing service.

### Mesopotamia Nursing Service.

Mr. Forster stated that the Army Order which grants Army of Occupation bonus to the nursing services is applicable to Mesopotamia.

### Pensioners Awaiting Hospital Treatment.

The Minister of Pensions stated that at present there are about 130 discharged disabled men awaiting hospital treatment as bed cases in the London area. The surgical cases will be admitted forthwith, and other cases with as little delay as possible.

### Demobilisation of Medical Officers.

Lord Cavendish-Bentinck asked the Secretary of State for War to explain some apparent inconsistencies in the relative terms of medical officers joining the Army before and after the Military Service Acts. Mr. Churchill said that many of these officers who joined voluntarily before the passing of the Acts, and who afterwards came under the provision of those Acts, are still of necessity retained on military service, and some are receiving less than those who have undertaken to serve for a further period of six months. A considerable number of medical officers with comparatively short service have from time to time been demobilised for special reasons, more particularly in order to meet the urgent needs of the civil population. Medical officers are now being demobilised in order of priority, having regard to length of service, age, etc., immediately they can be spared. Mr. Churchill regretted that it is not possible to extend the terms given to officers who bind themselves for a definite period to those who will not undertake this obligation.

